# Stable colonization of the kissing bug *Rhodnius prolixus* by *Trypanosoma cruzi* Y strain

**DOI:** 10.1371/journal.pntd.0012906

**Published:** 2025-03-12

**Authors:** Ruby E. Harrison, Kevin J. Vogel, Ronald Drew Etheridge

**Affiliations:** 1 Center for Tropical and Emerging Global Diseases, University of Georgia, Athens, Georgia, United States of America; 2 Department of Cellular Biology, University of Georgia, Athens, Georgia, United States of America; 3 Department of Entomology, University of Georgia, Athens, Georgia, United States of America; Centro de Pesquisa Gonçalo Moniz-FIOCRUZ/BA, BRAZIL

## Abstract

*Trypanosoma cruzi* is a single-celled eukaryotic parasite responsible for Chagas disease, a major cause of morbidity and mortality in Central and South America. While the host-pathogen interactions of *T. cruzi* have been extensively studied in vertebrate models, investigations into its interactions within its insect host remain limited. To address this gap and establish a genetically tractable system for studying parasite-vector dynamics, we conducted quantitative kinetic infection studies using the Y strain of *T. cruzi* and the model vector *Rhodnius prolixus*. We began by comparing parasite infection kinetics from two genetically diverse strains of *T. cruzi,* Brazil and Y, and demonstrated that ingested parasites from both strains transiently expand in the anterior regions of the insect digestive tract with stable colonization occurring in the hindgut over the long term. Notably, we demonstrated that the clonal Y strain, contrary to previous reports, can effectively infect and persist across multiple developmental stages of *R. prolixus*. Additionally, comparison of movement of parasites versus inert fluorescent microspheres introduced into artificial blood meals suggests that *T. cruzi* colonization of the *R. prolixus* gut occurs passively through peristaltic movement during digestion, rather than through active parasite-mediated chemotaxis. These findings highlight the *T. cruzi* Y strain - *R. prolixus* model system as a promising tool for the in-depth molecular characterization of parasite-vector interactions, potentially offering new insights into the biology of this neglected and deadly human pathogen.

## Author summary

The single-celled eukaryote *Trypanosoma cruzi* is a parasite which causes Chagas disease in humans, a condition that can result in debilitating enlargement of the esophagus and colon and which can additionally cause premature death by heart failure. *T. cruzi* is primarily transmitted to people from the feces of a blood-feeding insect vector known as a kissing bug or triatomine. Characterization of host-pathogen interactions of *T. cruzi* have largely focused on this parasite’s mammalian, rather than insect, hosts. However, a comprehensive understanding of how *T. cruzi* proliferates within kissing bugs is paramount to forming new strategies to disrupt transmission. In this study, we demonstrate that the Y strain of *T. cruzi* colonizes the major vector species *Rhodnius prolixus* and we characterize multiple aspects of parasite development within the insect digestive tract. We propose the *T. cruzi* Y strain - *R. prolixus* system as an ideal model for future interrogation of parasite-insect molecular interactions.

## Introduction

The causal agent of Chagas disease, *Trypanosoma cruzi*, is an obligate intracellular parasite that infects an estimated 6-7 million people in the Americas, with an at-risk population of 70 million [1]. Inoculation is followed by a brief acute stage of infection that, while mild or even asymptomatic, is also not completely cleared. While 70% of infected individuals progress to a clinically silent indeterminate stage, the remaining 30% often develop life-threatening pathologies [2,3]. No vaccine for *T. cruzi* infection is currently available and the only efficacious and approved treatment regimens are benznidazole and nifurtimox, chemotherapies which both consistently incur adverse side effects causing high drop-out rates [4] and which are estimated to be available to less than 1% of infected people [5]. Given the challenges in detection and treatment of *T. cruzi* infection, its status as an emerging pathogen in the U.S. is a serious public health concern [6].

Like several other protozoan parasites of humans, *T. cruzi* is primarily transmitted to humans by a blood-feeding arthropod vector. Competent *T. cruzi* vectors all belong to the insect subfamily Triatominae, colloquially called “kissing bugs”, although transmission congenitally, via blood transfusions, and from organ transplantation have also been reported [7]. Importantly, unlike the more common salivarian transmission strategy of other disease-causing trypanosomatids such as *T. brucei* or *Leishmania* species, *T. cruzi* is classified as a stercorarian (fecally transmitted) parasite [8]. During blood feeding, an infected triatomine will expel the contents of its hindgut onto its vertebrate host, releasing infectious flagellated non-dividing forms of the parasite known as the metacyclic trypomastigote in its excreta. If this stage of the parasite is able to enter the victim’s bite wound or nearby mucous membrane, it has the potential to invade any nucleated cell it encounters. Upon penetration of the host cell membrane and entrance into the cytosol, the metacyclic trypomastigote undergoes a dramatic morphological transformation into replicative amastigotes, a diminutive and immobile form of the parasite lacking a prominent flagellum. Following several days of proliferation and the eventual depletion of host cell resources, amastigotes initiate a stage transition whereby they reextend their flagella, again converting into infectious trypomastigotes which ultimately lyse the host cell and thus propagate the infection [9].

Repeated invasion and lysis of cells by *T. cruzi* over years or even decades leads to the destruction of host tissues, resulting in serious and sometimes fatal cardiomyopathies and megaviscera in patients with chronic Chagas disease [10]. Alternatively, following egress from an infected cell, circulating trypomastigotes have the potential to be ingested by a naïve triatomine insect. Trypomastigotes transition to the replicative extracellular epimastigote stage within the kissing bug digestive tract and rapidly multiply by binary fission. Only after reaching the intestinal hindgut and being exposed to certain environmental cues (e.g., low nutrients) will epimastigotes convert a final time to infectious metacyclic trypomastigote forms, and await release onto a new bite victim to begin the cycle anew [11].

Although the insect vector is central to ongoing transmission of this parasite, the scientific literature describing *T. cruzi-*host interactions has historically been heavily focused on mammalian infections [12–15]. Of the limited reports characterizing *T. cruzi* biology within its insect vector, comparatively few have investigated the molecular interactions between parasite and insect host. Little is known regarding how *T. cruzi* diverts nutrients, navigates and colonizes the vector gut lumen, interacts with the vector microbiome, and determines when and where to initiate life stage transitions into either replicative or infectious forms [16,17]. Much of our lack of understanding of the vector-parasite interactions can be attributed to inadequate resources to study this pathogen, and until recently, a lack of molecular tools to genetically manipulate *T. cruzi* or major triatomine vector species. Likewise, conducting longitudinal studies of *T. cruzi* development in kissing bugs presents several difficulties as it requires appropriate facilities for containment of infected arthropods and is additionally complicated by two major challenges of triatomine rearing: an exceptionally prolonged development period and a highly restricted diet of fresh vertebrate blood which must be provided via anesthetized animal or artificial membrane feeder [18,19]. A comprehensive characterization of the kinetics of *T. cruzi* infection within its vector remains limited, thus hindering our ability to accurately predict disease transmission dynamics and develop effective control strategies towards interrupting transmission.

Strains of infectious *T. cruzi* are characterized by genotype and specifically grouped into seven discrete typing units (DTUs) designated as TcI-VI and TcBat. While DTUs III-VI have been proposed or confirmed as hybrid lineages, DTUs I and II are confirmed clonal lines that diverged from a common ancestor 1-3 million years ago and which are responsible for the majority of chronic Chagas infections [20]. An attractive candidate strain of *T. cruzi* to begin interrogating trypanosome-kissing bug molecular interactions is the clonal Y strain (DTU II) as it has a recently sequenced and well-annotated genome [21] and is readily genetically manipulated using CRISPR/Cas9 genome editing [22–25]. However, the Y strain of *T. cruzi* is widely reported to only transiently colonize *R. prolixus,* rapidly disappearing from the gut by two weeks post-infection or earlier [26–28]. In contrast, other *T. cruzi* strains including Dm28c (DTU I) and CL Brener (DTU VI) have consistently been found to efficiently infect *R. prolixus* under experimental conditions with the majority of the parasite population residing in the hindgut [29,30]. Infected *Rhodnius* sp. collected from their natural habitats are dominantly colonized by DTU I genotypes [31,32]. Whether this observed association is because *Rhodnius* species are uniquely permissive vectors for DTU I *T. cruzi* strains, or is simply due to geographic overlap of these organisms, remains unclear.

Foundational studies demonstrated that the failure of the *T. cruzi* Y strain to infect *R. prolixus* was due to the susceptibility of epimastigotes to lysis by the bacterium *Serratia marcescens* [26,33]*,* an opportunistic pathogen which is a common resident of the gut microbiota of several hematophagous insects [34]. Comparative bioassays assessing trypanolytic activity of multiple *S. marcescens* strains further revealed that only prodigiosin (pigment) -producing strains eliminated *T. cruzi* populations *in vitro* while non-pigmented strains had no adverse effects on parasites [33]*.* In-house surveys of the enteric bacteria colonizing juvenile and adult individuals from our *R. prolixus* laboratory culture via 16S rRNA sequencing paired with culture-based methods revealed only sporadic presence of a non-prodigiosin producing strain of *S. marcescens* and indicated no pigment-producing *S. marcescens.* We therefore reasoned that individuals from our *R. prolixus* colony should support *T. cruzi* Y strain infection and tested this hypothesis with a series of straightforward assays comparing outcomes of infections using the model Brazil strain [20].

Using a quantitative real time PCR-based (qPCR) approach, we examined the kinetics of parasite expansion and colonization of three major regions of the triatomine insect gastrointestinal (GI) tract across time and comparing Y (DTU II) versus Brazil (DTU I) strain infections. We observed that Y strain parasites readily colonize our laboratory colony of *R. prolixus* in a manner indistinguishable from Brazil strain infection. We tracked a number of distinct variables with regards to the infection kinetics in our comparison and found that the number of parasites consumed during initial infection, the size of the insect at infection or the frequency of feeding prior to infection had little effect on the final levels of *T. cruzi* parasitemia. Ingested epimastigote-stage Brazil and Y strain *T. cruzi* progressed to infectious metacyclic forms released in triatomine feces, indicating parasites from both strains successfully completed their life cycle in *R. prolixus.* Focusing on the Y strain exclusively, we demonstrated that both epimastigotes and *in vitro* culture-derived blood stream trypomastigotes display equivalent infection kinetics and using fluorescent microspheres administered during the bloodmeal, we found that colonization of the vector intestinal tissues proceeds passively due to normal gut peristalsis. Finally, we observed that Y strain parasites also persist in *R. prolixus* juveniles and can be detected following successive developmental molts, likely resulting in lifelong infection of the vector.

## Methods

### Ethics statement

No vertebrate animals were used for the experiments described in this paper. For maintenance of the *R. prolixus* culture, insects were blood-fed using artificial membrane feeders following a standard procedure approved by the University of Georgia Biosafety Committee under protocol 2021-0057.

### Insects

*Rhodnius prolixus* were originally acquired from the laboratory of Ellen Dotson at the Center for Disease Control and Prevention in Atlanta, GA through BEI Resources. Insects were maintained in a climate-controlled incubator (Percival I-36VL) kept at 28°C and 70% relative humidity following a 12h:12h day/night cycle. Adults, fifth instar nymphs, and fourth instar nymphs were separated by life stage and maintained at densities of 50-100 individuals per cage while first, second, and third instar nymphs were housed together at densities of approximately 100-200 individuals per cage. Cages were either 1L volume polypropylene containers (Thermo Scientific Nalgene 2118-0032) or 32 oz plastic tubs (Choice 127DM32BULK) fitted with donut lids with snap-on clips (BugDorm BDC0003_12P). Both cage styles were topped with secured nylon mesh to allow blood feeding and gas exchange and contained vertical corrugated cardboard supports (Uline S-2714) for insects to climb on, to absorb excreta, and to serve as oviposition substrates. All *R. prolixus* life stages were offered defibrinated sheep blood (Hemostat Labs DSB800) on a weekly basis for a duration of 3-4 h at 25°C in the dark. The bacterial symbiont *Rhodococcus rhodnii* was added to blood at 10^6^ CFUs/mL to promote efficient development and high fecundity [35]. Blood meals were provided using glass water-jacketed artificial feeders (ChemGlass CG-1836-75) warmed to 37°C using a hot water bath and recirculating pump and using powder-free latex gloves (VWR 76319-672) as membranes.

### Parasites

Y strain *T. cruzi* was obtained from the American Type Culture Collection (ATCC) (50832) while Brazil strain *T. cruzi* were obtained from BEI Resources (NR-53932). Both strains were continuously maintained in culture *in vitro* as epimastigotes in liver infusion tryptose broth (LIT medium) [36] supplemented with 15% fetal bovine serum (FBS) which was first heat-inactivated for 1.5 h at 76°C. Trypanosomes were stored at 28°C in the dark in 12.5 cm^2^ cell culture flasks (VWR 10062-870), passaged every 2-4 d, and maintained at densities of 1×10^6^ - 4×10^7^ as determined using a particle and cell counter (Beckman Coulter Z2 8043-30-0016).

### Verification of *T. cruzi* strain identity

Genomic DNA was isolated from pellets of 10^7^ epimastigote-stage Brazil or Y strain parasites using the classic phenol/chloroform phase extraction method. DNA concentration was measured using a spectrophotometer and templates diluted to 10 ng/µl. Primer sets that exclusively amplify sequences from each DTU, originally developed by Munoz-San Martin *et al.* [37], were used in separate reactions each following identical conditions. Primer sequences and predicted amplicon sizes are listed in S1 Table. Reactions of 25 µl total volume each contained: 12 µl sterile water, 5 µl Q5 polymerase buffer (5x), 3.125 µl of each primer (5 µM), 0.5 µl dNTPs (10 mM), 0.25 µl Q5 high-fidelity polymerase (New England Biolabs), and 10 ng template DNA. PCR conditions consisted of an initial denaturation at 98°C for 1 min followed by 28 cycles of denaturation at 98°C for 30 sec, annealing at 70°C for 30 sec, and extension at 72°C for 30 sec, culminating in a final extension at 72°C for 1 min. PCR products were electrophoresed in 2% agarose at 60 V for 1.5 h.

### Infection of *R. prolixus*

*T. cruzi* epimastigotes were cultured as described above and were strictly maintained in log phase growth (5×10^6^ – 1.5×10^7^) continually for at least 5 d prior to infectious feeds. *T. cruzi* blood stream trypomastigotes were generated *in vitro* by infecting Human Foreskin Fibroblasts (HFFs) (generously provided by Dr. Jessica C. Kissinger, University of Georgia) with stationary-phase, maximum density epimastigotes kept in LIT medium for 7 d without passaging to induce metacyclogenesis [38]. For three days following trypanosome infection, HFFs were maintained at confluency in Dulbecco’s Modified Eagle’s Medium (DMEM) high glucose supplemented with 10% cosmic calf serum (CCS, VWR 16777-244). The medium was then decanted on day 4 post-infection and replaced with DMEM high glucose supplemented with 1% CCS. Trypomastigote- and amastigote-stage trypanosomes resulting from this infection were maintained in the same flask of HFFs for 2 weeks to amplify the parasite population.

Parasite density in culture medium was determined the day of insect infection either by particle counter (epimastigotes) or by manually counting using a Neubauer hemocytometer (blood stream trypomastigotes). Epimastigotes or blood stream trypomastigotes were isolated from LIT medium or infected HFF supernatant, respectively, by centrifugation at 1000 × *g* for 10 min. Parasites were washed 3 times in Hank’s Balanced Salt Solution (HBSS) to remove all traces of antibiotics from the culture medium and finally resuspended in defibrinated sheep blood which had been decomplemented by heating the serum fraction at 56°C for 1 h. The density of parasites used for infectious blood meals varied depending on the experiment and is specified in the pertinent Results sections.

Two to three days before infectious feeds, adult-stage *R. prolixus* were marked with two colors of nail polish on the dorsal side of the thorax to permit identification and tracking of individuals. Infectious blood meals were offered to insects using the artificial membrane feeders described above (Insects) for 3 h at 25°C in the dark. The blood meal mass consumed by each individual insect was determined by calculating the difference between its total mass (Denver Instrument Company Analytical Balance A-160) 1 d prior to the feed versus its mass at 3-5 h post-blood meal, a time point well after this species completes diuresis of excess blood meal-derived fluid [39] to avoid inter-individual fluctuations in mass. First instar nymphal *R. prolixus* could not be marked with nail polish, as this later induced molting failure, nor accurately weighed as individuals due to their negligible mass. Average blood mass consumed by nymphs therefore was estimated by weighing several cohorts consisting of 10 pooled nymphs to generate an average mass of non-fed individuals followed by weighing 10 pooled insects at 3-5 h post-blood meal to generate an average replete nymph mass. Hence, blood mass consumed by adults was directly measured, whereas blood mass consumed by nymphs was estimated by comparing averages of randomly selected cohorts.

Following infection, non-fed individuals were separated and discarded. Replete adults were housed in groups of 30-50 per cage and both parasitemia and mortality tracked weekly until the experiment was completed. Replete nymphs were housed in groups of 75-100 per cage until one week prior to expected onset of molting, at which time they were transferred to 24-well (1^st^-2^nd^ instars) or 12-well (3^rd^-4^th^ instars) tissue culture plates to allow precise tracking of development. Nymphs were sampled to quantify parasitemia at 1d post-infection (1^st^ instars) or 7d post-molt (all ensuing instars). As bugs housed individually did not readily blood feed, nymphs that had molted were again pooled in cages and offered a non-infectious blood meal at 7-14 d post-molt. To minimize variation, only synchronous cohorts of nymphs that molted within a 4-day time span were pooled and offered a subsequent blood meal to allow development to the next life stage. Nymphs that molted earlier or later than this 4-day window were discarded.

### DNA isolation and quantitative real-time polymerase chain reaction

*T. cruzi-*fed *R. prolixus* were anesthetized at 4°C for 30 min, then appendages removed using small spring scissors (Fine Science Tools 91500-09) and whole digestive tract (comprising the anterior midgut (AM), posterior midgut (PM), and hindgut (HG)) explanted into ~200 µl phosphate-buffered saline (PBS 1x) using needle-nosed forceps (Fine Science Tools 11251-10). Malpighian tubules, tracheae, and fat body tissue adhered to the digestive tract were removed and the gut precisely separated into the AM, PM and HG regions using a disposable scalpel. Each severed gut region was transferred with forceps to a microcentrifuge tube containing 5 µl Proteinase K 20 mg/ml (Thermo Fisher) and 200 µl SNET lysis buffer (400 mM NaCl, 1% w/v SDS, 20 mM Tris-Cl pH 8.0, and 5 mM EDTA pH 8.0; Cold Spring Harbor Protocols). Five to seven 1.7 mm stainless steel beads were added (Next Advance SSB16) per tube and tissues were macerated for 2 min using a bead homogenizer (Next Advance Bullet Blender BBX24). Tissues were then incubated at 56°C for 2 h to facilitate Proteinase K digestion. Total DNA was isolated from tissue lysates using phenol:chloroform:isoamyl alcohol 25:24:1 (Fisher Scientific BP1752I-400) according to the manufacturer’s instructions. DNA suspended in the aqueous phase was precipitated by addition of sodium acetate 3 M pH 5.2 and isopropyl alcohol followed by centrifugation at 15,000 × *g* for 30 min at 4°C. DNA pellets were washed once in 75% ethanol, solubilized in 30 µl nuclease-free sterile water, and integrity assessed by spectrophotometer (Biotek Epoch).

DNA templates were used to quantify total parasite genome copies per tissue by real-time PCR (absolute expression) using primers TczF (5’ - GCTCTTGCCCACAMGGGTGC - 3’) and TczR (5’ - CCAAGCAGCGGATAGTTCAGG - 3’) which target a 195 bp microsatellite tandem repeat DNA region in *T. cruzi* [40]. The number of repeats targeted by these primers is circa 120,000 in *T.* cruzi [41]. The standard curve for real-time PCR quantification was not generated by cloning the target fragment into a plasmid backbone. Instead, total DNA was extracted from 10^7^ log-phase *T. cruzi* Y epimastigotes counted in triplicate by particle counter and qPCR performed on decimal serial dilutions ranging from 10^1^-10^6^ parasite diploid genome copies which yielded a standard curve of R^2^ = 0.998, E = 74%, slope = -4.156, and y-intercept = 29.565. Reactions were performed in four technical replicates and contained 1 µl undiluted DNA template, 0.5 µl Fd/Rv primer mix (5 µM each), 3.5 µl nuclease-free H_2_O, and 5 µl iTaq™ SYBR Green Supermix (Bio-Rad 1725121). qPCR was carried out using a CFX96 Real-Time System (Bio-Rad) following a 3-step program: 95°C for 10 min followed by 35 cycles of 95°C for 10 sec, 55°C for 15 sec, and 72°C for 20 sec and ending with a melt curve analysis (ramp from 72°C to 95°C during 1 min) to confirm amplification of a single product.

### Flow cytometry

Adult *R. prolixus* starved for 3 weeks were fed defibrinated sheep blood containing 50 µl/ml (= 2.84 × 10^9^/ml) Fluoresbrite Polychromatic Red Microspheres, 2.0 µm diameter (Polysciences 19508-2) with or without 1 × 10^7^/ml *T. cruzi* Y strain epimastigotes (infected/uninfected treatments, respectively) using the artificial membrane feeder system described above. Replete individuals were separated post-meal and 6 individuals per treatment were sampled every 24 h by explanting the digestive tract and macerating its three principal regions (AM, PM and HG) in 200 µl PBS using a bead homogenizer. AM, PM and HG homogenates were diluted 1:10 in 500 µl PBS and number of red particles in a 30 µl sample volume quantified using the propidium iodide detection channel on a NovoCyte Quanteon laser flow cytometer (Agilent). FlowJo software (https://www.flowjo.com/) was used to analyze the resulting data. The total sum of red particles in the AM+PM+HG from each individual insect was calculated to generate the relative proportion of red microspheres in each gut region compared to the entire GI tract as a whole.

### Data analysis

For all assays, individual bugs or tissues explanted from individuals served as the unit of biological replication. Prior to analysis, data sets were assessed for (a) Gaussian distribution of residuals using the Shapiro-Wilk test and (b) homogeneity of variances using either the F test of variances where there were two treatments or Bartlett’s test where there were three or more. For two treatments, size of blood meal mass was compared either using a two-tailed unpaired Student’s t-test or a Mann-Whitney U nonparametric test. For three treatments, size of blood meal mass was compared using either one-way analysis of variance (ANOVA) followed by Tukey’s multiple comparisons or Kruskal-Wallis test followed by Dunn’s post-hoc. Proportions of bugs to feed to repletion were compared among treatments by contingency table analysis using either Fisher’s exact test or Chi-square test. Pearson correlation analysis was used to compare mass of blood ingested by bugs with ultimate whole-body parasitemia at the time of sampling. Survival (adult *R. prolixus*) and molting (juvenile *R. prolixus*) curves were compared among treatments using Mantel-Cox analysis. Finally, parasitemia in insect gut regions over time was compared among treatments using a linear repeated measures mixed effects model in which the individual insect sampled was assigned as a source of random variation. Data analyses were performed using R v4.2.2 using the nlme package for linear mixed effects models. Data were graphed using GraphPad Prism v.9.0.1. and final figures assembled using Adobe Illustrator v.23.0.1.

## Results

### Y strain *T. cruzi* stably infects *R. prolixus* with kinetics analogous to the Brazil strain

**Fig 1:**
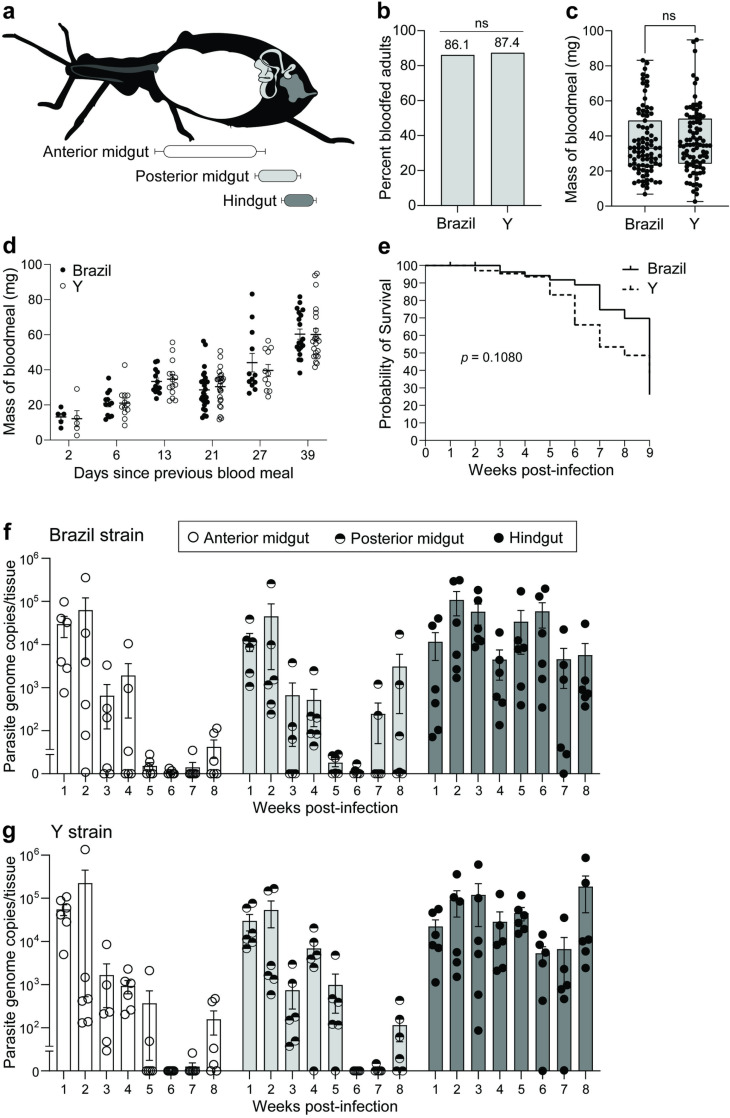
The Y strain of *Trypanosoma cruzi* stably infects the vector *Rhodnius prolixus.* (a) Diagram indicating the three major regions of the kissing bug digestive tract in which parasite populations were quantified. (b) Bars indicate the total percentage of adults that consumed infectious blood, which did not significantly differ between treatments (Fisher’s exact test). (c) Adult *R. prolixus* consumed blood meals that did not differ significantly in mass when the blood contained Brazil strain versus Y strain *T. cruzi* (Mann-Whitney U-test). Dots representing blood meal masses of individual bugs overlay box-and-whisker plots indicating data quartile and median values. (d) Six cohorts of adult *R. prolixus* were used for *T. cruzi* infection which had been starved for different durations before the experiment. Regardless of which strain of *T. cruzi* insects were exposed to, insects starved for longer time periods consumed larger blood meals. (e) Survival curves of Brazil- and Y-infected adult *R. prolixus* over a nine-week period of starvation following the initial infectious feed. No difference was detected in the probability of survival between treatments using a Mantel-Cox (log rank) test. (f, g) Absolute quantitative PCR of total DNA isolated from portions of the gut of *R. prolixus* indicated that populations of (f) Brazil and (g) Y strain *T. cruzi* diminish in anterior and posterior midgut regions, but stably persist long-term in the insect hindgut. Dots representing total parasite genome copies per tissue from individual insects overlay bars showing mean and standard error. A linear repeated measures mixed effects model examining interactions of treatment × tissue × time on parasite load which additionally assessed individual insects sampled as a source of random variation indicated no significant differences between prevalence of Brazil versus Y strain *T. cruzi* in *R. prolixus*.

The specific region(s) of the gut in which *T. cruzi* blood stream trypomastigote forms convert to dividing epimastigote stages has historically been contested, with early studies documenting this in the stomach-like anterior midgut but with more recent studies suggesting this either occurs in the posterior midgut or the hindgut [42]. We tracked the kinetics of *T. cruzi* colonization in three morphologically and functionally distinct regions of the triatomine digestive tract: the anterior midgut (AM), in which ingested blood is stored; the posterior midgut (PM) (historically referred to as the intestine), where digestion occurs; and the hindgut (HG) (historically referred to as the rectum), where fecal matter accumulates (see diagram in Fig 1a). We additionally compared infection kinetics of *T. cruzi* from two different laboratory strains, the Y strain (DTU II) and the Brazil strain (DTU I), the genotypes of which we confirmed by PCR amplification of variable regions within highly conserved genes (S1 Fig). Adult stage *R. prolixus* were provided 5×10^6^ epimastigote-stage *T. cruzi* per milliliter (ml) of defibrinated and decomplemented sheep blood using an artificial membrane feeding system (see Methods) and were weighed before and after infection to determine the mass of blood ingested.

The proportion of *R. prolixus* individuals to feed to repletion on infectious blood was not statistically different between the two strains by Fisher’s exact test (two-sided, *p* = 0.3766) (Fig 1b). Adult insects consumed blood meals that did not significantly differ between treatments by Mann-Whitney U test (two-tailed, *p* = 0.6110) but which varied considerably within treatments (Fig 1c). This appeared to be primarily influenced by the length of the starvation period prior to the infectious meal (Fig 1d). Longevity for Brazil- versus Y-fed *R. prolixus* was also not significantly different over an extended time course of 9 weeks following infection, during which insects were not offered any subsequent blood meals (Mantel-Cox test, Χ^2^ = 2.584, df = 1, *p* = 0.1080) (Fig 1e). We used quantitative real-time PCR (qPCR) to assess *T. cruzi* genome copies in total DNA extracts of *R. prolixus* gut regions following an 8-week time course. As previously reported for CL Brener and Dm28c strain kinetics in *R. prolixus* [29,30], the Brazil strain of *T. cruzi* transiently appeared in the insect anterior and posterior midgut regions but maintained a stable infection of the triatomine hindgut throughout the course of the study (Fig 1f). Importantly, we found that the Y strain of *T. cruzi* was able to successfully colonize *R. prolixus* adults and that the parasites persisted in high numbers in the gastrointestinal (GI) tract despite the starvation-associated mortality of their hosts (Fig 1g). Analysis using a repeated measures mixed effects linear model indicated that overall, there was no significant strain-dependent effect on parasite burden in *R. prolixus* (*p* = 0.1242)*.* Additionally, this model indicated no significant interactions of strain × tissue (*p* = 0.1656) or strain × sampling time (*p* =0.3669).

**Fig 2:**
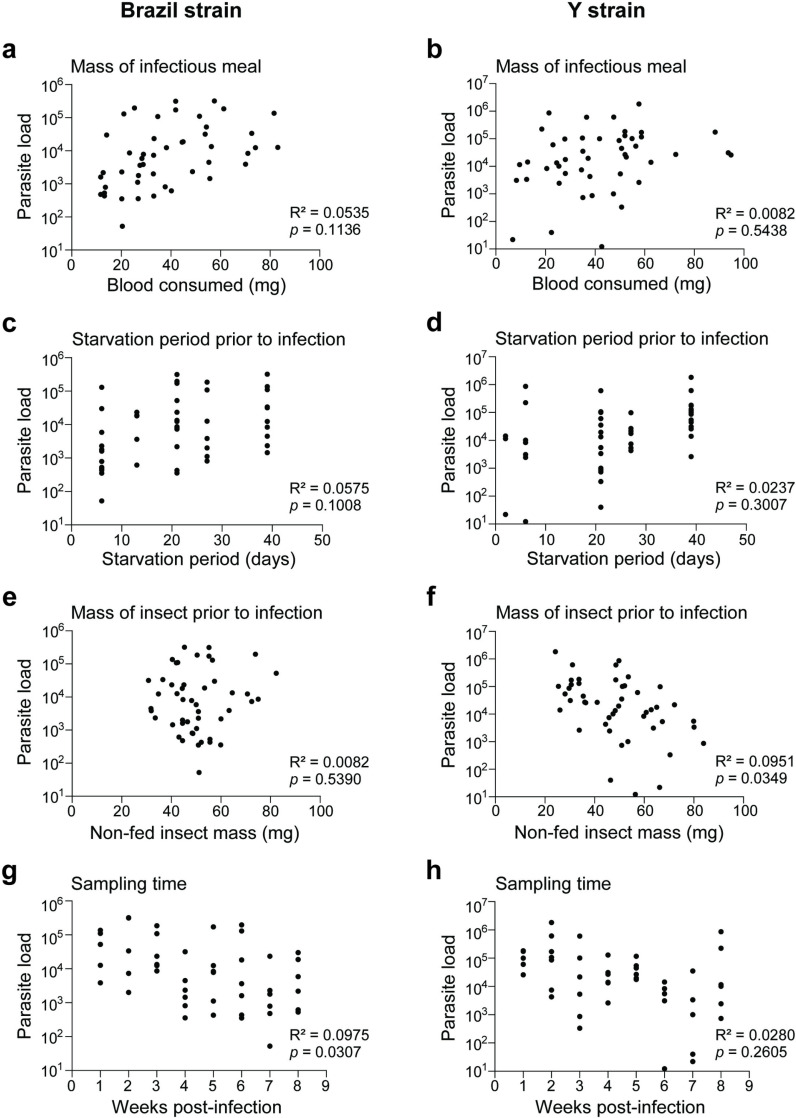
Parasite load is not consistently correlated with *R. prolixus* nutritional status or with duration of infection. Correlations of parasite load for Brazil-infected (left column) versus Y-infected (right column) adult *R. prolixus* compared to: mass of the infectious blood meal (a, b), starvation period of the insect prior to *T. cruzi* infection (c, d), mass of the starved insect prior to infection (e, f), and duration of infection prior to sampling (g, h). Scatterplots show values for individual insects comparing whole gut (AM+PM+HG) parasite load on the *x*-axis versus indicated values on the *y*-axis. Results of Pearson correlation coefficient analysis (Pearson *r* and *p*-value) are indicated.

*R. prolixus* exhibited high variation in parasite load regardless of the strain of *T. cruzi* used for infection and, as a result, we examined what factors could be driving this variability. A fundamental parameter of infection assays at the cellular or organismal level alike is how the ratio of infectious particles to target(s) influences the outcome of infection. Vertebrates with acute-phase *T. cruzi* infection commonly present with circa 5×10^4^ parasites per ml blood, but typical triatomine infection assays use 1-5×10^7^ parasites per ml blood [17]. We therefore compared the size of the infectious blood meal consumed by individuals, a direct indicator of the number of parasites ingested, to the whole-GI tract parasite load on the day of sampling using Pearson Correlation analysis. Surprisingly, the mass of the infectious blood meal was not significantly correlated with parasite load for either Brazil or Y-infected bugs (R^2^ and *p*-values indicated on graphs) (Fig 2a and 2b). We secondly examined the length of starvation period for insects prior to infection as a potential driver of *T. cruzi* success given both vector and parasite likely compete for limiting nutrients derived from the blood meal [42]. We again found that the duration of prior starvation did not significantly affect parasitemia of either strain (Pearson Correlation analysis) (Fig 2c and 2d). Comparing the whole-body mass of non-fed insects prior to infection showed no relationship to parasite load for Brazil-infected bugs, while Y-infected *R. prolixus* showed a significant but modest relationship to parasite burden in which lower body mass adults were more heavily infected (Pearson Correlation analysis) (Fig 2e and 2f). Finally, we expected that later sampling time points would be correlated with lower parasite loads because insects were starved following the initial infection and we predicted the lack of additional nutrients would negatively impact *T. cruzi*. While *R. prolixus* showed this general trend for both treatments, the relationship was only statistically significant for Brazil-fed adults (Pearson Correlation analysis) (Fig 2g and 2h).

### Y strain parasites infecting *R. prolixus* convert to metacyclic trypomastigotes that are excreted during ingestion of subsequent blood meals

Studies reporting the inability of Y strain *T. cruzi* to colonize *R. prolixus* indicate ephemeral presence of epimastigotes for a few days in the digestive tract before disappearance [26–28,43,44], while screening of insect excreta (urine/feces) for parasites five weeks following infection showed few or none were present [44]. Since the Y strain was able to colonize our culture of *R. prolixus* however, we next asked whether it was also able to complete its normal life cycle transition into infectious metacyclic trypomastigotes within this species. We examined excreta from a stably infected cohort of 20 adult insects following several refeeds of non-infectious blood meals. We observed that the numbers of metacyclic trypomastigotes increased with subsequent blood meals, with substantially more parasites being released from Brazil-infected compared to Y-infected *R. prolixus* (Table 1). This suggested that the Y strain is able to both effectively colonize and complete its life cycle in this vector.

**Table 1:** Presence of metacyclic trypomastigotes in expelled feces of Brazil and Y strain *T. cruzi-*infected *R. prolixus.* Insects pooled in cohorts of 20 were provided additional non-infectious blood meals biweekly following initial infection with 5 × 10^6^ epimastigotes/mL blood. Excreta were collected and metacyclic forms quantified using a Neubauer hemocytometer and an inverted microscope. Following blood meal 2, feces from adults infected with Brazil or Y strains each showed one drop-like epimastigote and one metacyclic form. Following blood meal 3 and all subsequent meals, metacyclic trypomastigotes were exclusively observed in excreta.

Blood meal	Brazil strain	Y strain
2	2	2
3	2,000	30
4	47,450	3,200
5	75,000	7,500

### Time-resolved tracking of *T. cruzi* populations reflects the prevalence of living parasites in the insect vector gastrointestinal tract

**Fig 3:**
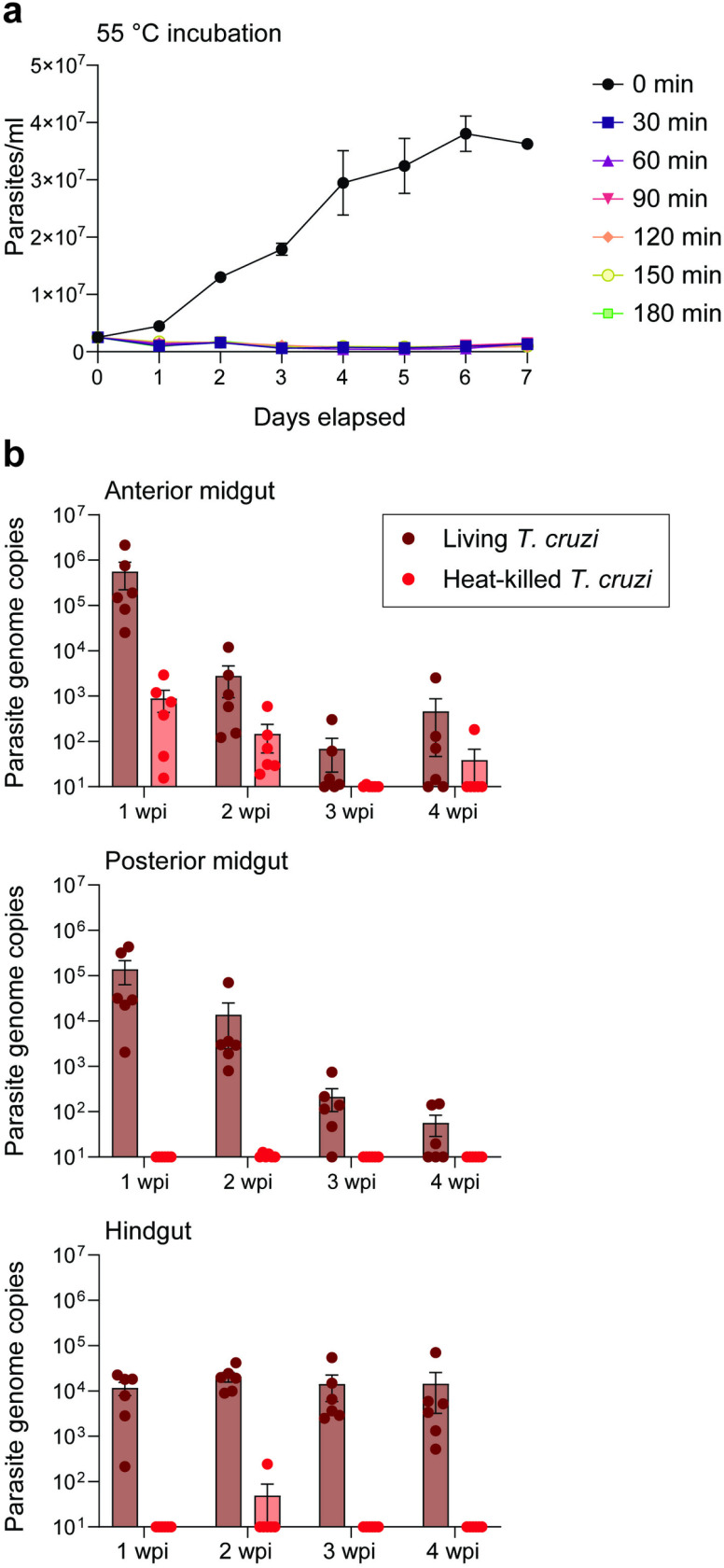
DNA from heat-killed *T. cruzi* does not persist long-term in the gut of *R. prolixus.* (a) Incubation of *T. cruzi* Y strain epimastigotes at 55°C for any period of time ranging from 30-180 minutes fully inhibited parasite proliferation compared to non-heat-treated controls, as quantified daily for one week by Coulter particle and cell counter. (b) Parasite burden detected by qPCR in insect tissues from *R. prolixus* fed living, intact *T. cruzi* Y strain epimastigotes versus *R. prolixus* fed dead, heat-killed epimastigotes (bottom). Dots representing total parasite genome copies per tissue sample overlay bars showing mean and standard error.

Our analysis of parasite load using PCR-based detection of *T. cruzi* relies on the assumption that we are only detecting living parasites. However, our methodology does not strictly distinguish between these two possibilities. To ensure that our qPCR approach accurately reflects parasitemia of viable parasites, we assessed the persistence of target DNA when *R. prolixus* was fed heat-killed parasites. We incubated Y strain epimastigotes at 55°C for increasing 30-minute intervals up to 180 minutes maximum, returned them to normal culture conditions, and monitored growth for one week to ensure the heat treatment permanently stopped parasite proliferation. We observed that even the briefest 30-minute treatment totally ablated parasite viability and they were unable to recover growth in *in vitro* culture; longer incubations at 55°C produced an identical effect (Fig 3a). Both living and heat-killed (55°C, 30 min) parasites were fed to adult *R. prolixus* and insects were assayed each week for one month to assess how long parasite DNA persists in the vector. qPCR of *R. prolixus* gut regions showed that, in contrast to insects fed an equivalent titer of living parasites (Fig 3b), DNA from dead epimastigotes completely disappears from the anterior midgut after two weeks and is only rarely detected in the posterior midgut and hindgut at very low levels.

### Y strain *T. cruzi* epimastigotes and blood stream trypomastigotes are both infectious to *R. prolixus*

**Fig 4:**
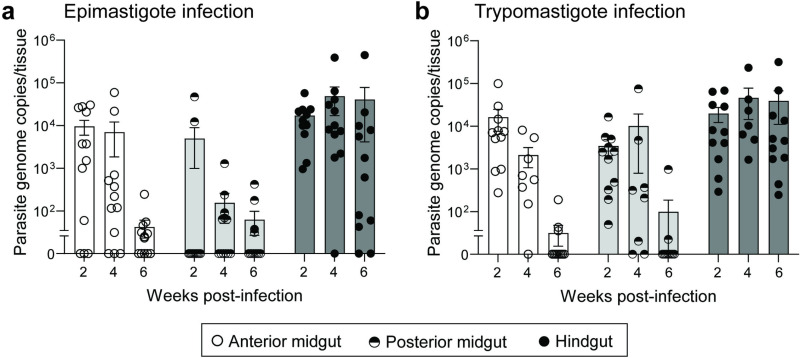
Epimastigote and trypomastigote stages of *T. cruzi* Y strain are both infectious to *R. prolixus.* (a, b) *T. cruzi* epimastigotes and blood stream trypomastigotes cultured *in vitro* are both infectious to *R. prolixus* and follow similar kinetics of colonization in the insect digestive tract. Dots representing total parasite genome copies per tissue from individual insects overlay bars showing mean and standard error.

In natural settings, triatomines are infected following ingestion of low numbers of blood stream trypomastigote forms circulating in a vertebrate host. While our prior observations established that epimastigotes are able to initiate infection, we asked if the kinetics of infection and tissue colonization differed when the vector was infected with blood stream trypomastigotes. We compared *R. prolixus* colonization by Y strain *T. cruzi* epimastigotes versus blood stream trypomastigotes cultured *in vitro* and determined that both show highly similar kinetics in the insect digestive tract and plateau at roughly equivalent populations of 10^4^-10^5^ in the hindgut (Fig 4a and 4b). One difference between treatments was elevated levels of parasites from blood stream trypomastigote infection in the posterior midgut during the first four weeks post-blood meal. However, colonization of this region of the gut for both treatments equivalently dropped to zero in most individuals by six weeks post-infection. Analysis using a mixed effects repeated measures linear model indicated no significant differences in overall parasite load between treatments (*p* = 0.8973) and no significant difference in the interaction of treatment × tissue (*p* = 0.9342). Since the colonization kinetics of the *R. prolixus* hindgut were highly similar following infection using epimastigotes versus blood stream trypomastigote stages, we moved forward using epimastigotes for ensuing infection assays due to their superior ease and reproducibility in *in vitro* culture.

### Y strain *T. cruzi* colonize and transstadially persist in *R. prolixus* juveniles

**Fig 5:**
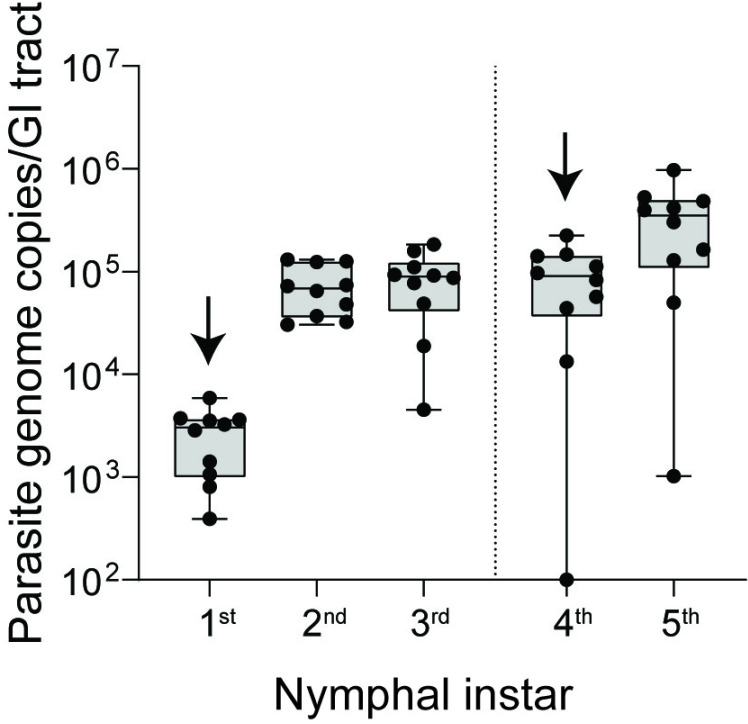
Y strain *T. cruzi* infects and persists in *R. prolixus* nymphs following repeated molts. Arrows indicate nymphal stage at which infectious meal was initially provided. Dots representing total parasite genome copies per nymph (whole gut) overlay box-and-whisker plots showing median and quartile values.

Juvenile stages of triatomines require a blood meal during each nymphal instar to obtain the necessary nutrients for development. Each blood meal is, therefore, an opportunity to acquire *T. cruzi* and all stages of *R. prolixus* are susceptible to infection with this parasite [16]. In nymphal triatomines, parasites face an additional barrier to persistence in the gastrointestinal tract, however, because the lining of the hindgut is everted and shed in the final stage of ecdysis (molting) in each instar [45]. The prevailing hypothesis is that *T. cruzi* maintains a small population of dividing epimastigotes in the triatomine posterior midgut, which is not everted, and which may serve as a reservoir to repopulate the hindgut following molting [42]. We repeated epimastigote infection assays in nymphal *R. prolixus* to verify whether or not Y strain *T. cruzi* persists transstadially, i.e., following progressive molts. Nymphs infected at the first instar stage maintained high GI tract populations of *T. cruzi* that expanded with ensuing molts to the second and third instars; similarly, fourth instar nymphs maintained and increased parasite burden following molting to the fifth instar (Fig 5).

### Y strain *T. cruzi* colonize *R. prolixus* gut regions passively via natural peristalsis

**Fig 6:**
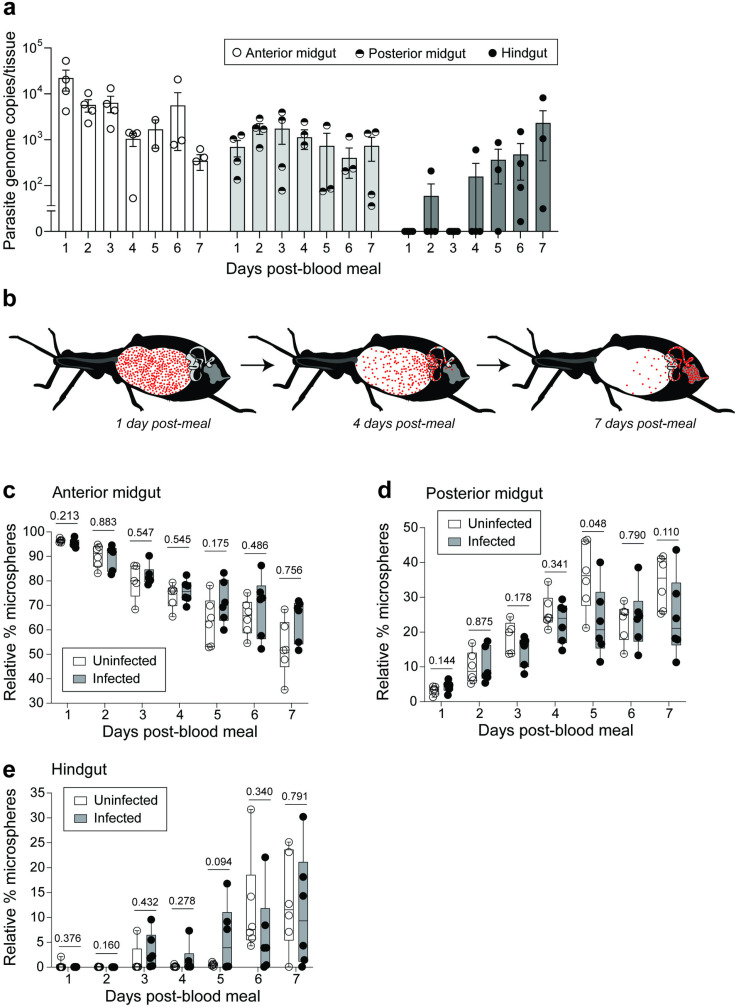
*T. cruzi* Y strain reaches the bug hindgut passively via peristalsis during blood digestion. (a) Results of qPCR indicating daily parasite burden in digestive tract tissues over the course of one week following infection. Dots representing total parasite genome copies per tissue from individual insects overlay bars showing mean and standard error. (b) Diagram illustrating passage of fluorescent beads (microspheres) from the anterior to the posterior regions of the insect gut as blood digestion progresses. (c-e) Box-and-whisker plots showing relative percent fluorescent microspheres in separate insect gut regions out of the total red particle counts for whole GI tracts; dots indicate values for individual insects with n = 6 per time point. Values are shown for *R. prolixus* fed microspheres in sterile blood alone (Uninfected) versus blood containing *T. cruzi* Y epimastigotes (Infected). P-values for pairwise t-tests comparing relative proportions of microspheres between uninfected and infected treatments are indicated for each time point.

Prior research has shown that various other protozoan parasites transmitted by insect vectors such as *Plasmodium spp., T. brucei,* and *Leishmania spp.* exhibit tissue tropism once inside their invertebrate host. *Plasmodium* ookinetes, for example, cross the mosquito midgut epithelium to reach the basal lamina [46]; *T. brucei* procyclic forms and *Leishmania* promastigotes travel from the vector midgut anteriorly to reach either the salivary glands or the stomodeal valve and pharynx, respectively [47–50]. Because *T. cruzi* epimastigote stages are motile and traverse the insect GI tract in order to take up residence in the hindgut, we asked if *T. cruzi* actively navigates to the hindgut and escapes the insect midgut environment in a manner similar to other eukaryotic parasites or if they instead are simply passively carried to the vector hindgut by the gut peristaltic movement during digestion. To begin assessing this, we infected *R. prolixus* adults with Y strain *T. cruzi* and conducted a high-resolution time course, sampling insect gut tissues each day for one week post-infection. We observed that parasites reach the posterior midgut by approximately 24 hours and the hindgut by 5-6 days post-infection (Fig 6a).

To investigate the mechanistic underpinnings of this process, we next fed adult insects inert red fluorescent microspheres (2.0 µm in diameter) which are similar in size to *T. cruzi*. We fed microspheres both alone and in combination with Y strain *T. cruzi* and assessed the rate of microsphere traversal through the GI tract. A diagram illustrating the passage of red fluorescent beads (microspheres) from the anterior to the posterior regions of the gut as blood digestion progresses is shown in Fig 6b. Following a blood meal containing microspheres, 6 insects were destructively sampled every 24 h for 7 days; for each individual, the GI tract was explanted, the AM, PM, and HG separated and homogenized in PBS, and the number of fluorescent particles in each homogenate quantified using flow cytometry. To highlight the progressive movement of microspheres down the GI tract, data are graphed as proportions in which the denominator is total fluorescent particles summed per whole GI tract for one individual and the numerator is fluorescent particles detected solely in the lumen of the AM (Fig 6c), PM (Fig 6d), or HG (Fig 6e). We additionally compared the movement of red microspheres through the GI tract in *R. prolixus* fed *T. cruzi* epimastigotes along with the microspheres (Infected) or not (Uninfected). The microspheres progressed passively through the intestinal tract at the same rate as the parasites, with the first substantial proportion of microspheres also appearing in the hindgut at 5-6 days (6e). While low levels of microspheres were detected slightly earlier in hindgut homogenates of infected individuals, there were no significant differences in relative quantity of microspheres between treatments as determined via pairwise comparisons of treatments using unpaired t-tests and correcting for false discovery rate using the two-stage step-up method of Benjamini, Krieger, and Yekutieli (*p*-values for each comparison indicated on graphs).

## Discussion

As part of our long-term goal of identifying parasite factors necessary for infection and colonization of the hematophagous triatomine vector by *T. cruzi*, our lab has focused on the model species *R. prolixus*. Although *R. prolixus* is only 1 out of over 140 triatomine species in the Americas [51] that transmit *T. cruzi*, it has been extensively studied in laboratory settings for almost a century. Since Sir Vincent Wigglesworth’s pioneering use of *R. prolixus* to examine insect physiology in the 1930s, this species has gained prominence for its ease of cultivation and utility in studying parasite-vector interactions with *T. cruzi*, culminating in its becoming the first non-dipteran insect vector to have its genome fully sequenced [52,53].

In parallel to developing this vector system, we have also worked to establish a genetically tractable model parasite strain that will allow us to carry out insect infection studies to identify factors important for this process. The introduction of CRISPR/Cas9 technology into *T. cruzi* has dramatically improved the speed and efficiency of molecular manipulation in this organism making epitope tagging and gene knockouts a routine practice [22,54]. Additionally, our lab has also developed the first conditional knockdown tool to study essential genes in this parasite, opening the door for us explore not only *T. cruzi*’s basic biology, but also begin identifying what specific parasite genes are crucial for the successful infection of both its insect and mammalian host for the first time [55]. It is important to note, however, that many of these molecular tools and techniques were developed for use in the Y strain (DTU II) of *T. cruzi*, which benefits from high transfection efficiency and an exceptionally well-annotated clonal genome [21].

In spite of the Y strain’s proven utility in a laboratory setting, there are only a few reports which have used this DTU (II) in *R. prolixus* infection studies [33,44]. What these few studies do highlight, however, is that the Y strain appears unable to effectively colonize and persist in *R. prolixus*. A body of evidence demonstrates this can be attributed to trypanolytic activity of prodigiosin-producing strains of the bacterium *Serratia marcescens*, initially observed *in vivo* in the insect host and further characterized *in vitro* [26,33,44,56,57]. More recently, factors from the hemolymph and saliva of *R. prolixus* were demonstrated to directly lyse Y strain but not DTU I strains of *T. cruzi in vitro* [44,58–60]. Given, however, that our insects do not harbor prodigiosin-producing *S. marcescens*, and given that the trypanolytic factors in *R. prolixus* hemolymph and saliva have not been demonstrated to enter the insect gut lumen where *T. cruzi* resides, we proceeded to determine if our Y strain and *R. prolixus* colony could serve as a model system for our studies of vector-parasite interactions.

We began by first establishing a reliable method to quantitatively track parasite infection in various intestinal regions of the vector across time. Early methods to detect and quantify *T. cruzi* in kissing bugs were restricted to visual examination of flagellates isolated from dissected and macerated triatomine guts via microscopy, and these methods remained the primary strategy for nearly a century [29]. While visual examination provides invaluable information concerning parasite morphology and life stage transition, *T. cruzi* is not easily separated from insect tissue debris or feces for clear observation; additionally, manual counts using a Neubauer hemocytometer are only accurate above concentrations of 10^4^/ml [29]. Using genetically manipulated parasites expressing fluorescent proteins or luciferase can improve the ability to detect the presence and location of viable *T. cruzi*, but these methods are either not quantitative or suffer from the light obscuring effects of the digested blood meal [29,61,62]. Within the last decade however, researchers have begun applying quantitative PCR (qPCR) methods to more accurately track parasite populations in insect and mouse tissues alike (reviewed in [30]). Hence, identification and quantification of trypanosomes in triatomine vectors via qPCR is increasingly becoming a standard practice in the field, and has the additional advantage of being applicable to field-caught specimens [63–65].

Using qPCR, we compared the infection kinetics of the well characterized Brazil strain (DTU I) to our Y strain (DTU II) and determined that both *T. cruzi* strains readily infected *R. prolixus* with comparable kinetics with both reaching equivalent levels of parasitemia in the hindgut. Intriguingly, older microscopy-based tracking of *T. cruzi* in various kissing bug species reported that primary parasite populations establish stable infections in the intestine-like posterior midgut (reviewed in [42]). This model suggests that the majority of *T. cruzi* parasites reside in the posterior midgut as proliferating epimastigotes, establishing a core population from which a subset of mammalian infectious metacyclics split off and reside in the hindgut in lower numbers as they await release onto the next bite victim [16,66]. Our data show this is not the case for Brazil or Y strains, which appeared to transiently traverse anterior gut regions and stably colonize the hindgut regions. While it is possible that epimastigotes remain in the midgut regions, these populations are a relatively small proportion of the total insect parasitemia at 8 weeks post-infection.

It is important to acknowledge that the concentrations of parasites used in our infectious blood meals are substantially higher (5 × 10^6^/mL) than the reported parasitemia of mice experimentally infected with Y strain *T. cruzi*, which typically peaks at around 1 × 10^6^/mL during acute phase infection and drops thereafter to circa 1-5 × 10^4^/mL or lower [67]. Infection studies of triatomines using artificial feeding systems routinely use 5 × 10^6^ - 5 × 10^7^ epimastigotes/mL blood as an inoculum, and we elected to follow this standard methodology when establishing baseline kinetics of Y strain in *R. prolixus*. Curiously, our study revealed that the initial number of parasites consumed does not correlate with later parasite burden, matching prior observations in experimental infection of nymphal and adult-stage *Triatoma infestans* [68]. Our results suggest that low numbers of parasites are sufficient to produce stable infections in kissing bugs: for instance, we found that individuals that consumed blood meals weighing under 10 mg and corresponding to less than 500 epimastigotes ended up with average parasite loads of 10^4^-10^5^, while individuals consuming close to 100 mg blood (5000 epimastigotes) had equivalent parasite burdens. The quick expansion of *T. cruzi* to a stable upper plateau in insects matches the rapid proliferation potential of epimastigotes *in vitro,* and the amplification of parasites from low numbers in triatomines was already long suspected given a) the low parasitemia of infected vertebrates during chronic-phase infection [69] and b) the high fidelity of xenodiagnosis as a diagnostic tool still in use in the medical field [70].

Y strain *T. cruzi* evinced no discernible difference in infection kinetics when epimastigotes versus blood stream trypomastigotes were used to infect *R. prolixus*, and also persisted transstadially in nymphs across multiple instar molts, resulting in lifelong infection of the insect. Despite similar infection kinetics and parasitemia, Y strain-infected *R. prolixus* produced notably fewer metacyclic forms compared to Brazil strain-infected insects. While significant, a potential pitfall of our methods was the need to pool feces from cohorts of infected individuals, necessitated by *R. prolixus* recalcitrance to blood feed in isolation or even in small numbers. Although the underlying reason for this difference in number of metacyclic trypomastigotes remains unclear, it is possible that DTU I strains are better adapted for transmission by *R. prolixus* and thus either have higher rates of *in vivo* metacyclogenesis or are more responsive to physiological cues provided by the insect leading to greater mobilization and release during a bloodmeal. It is also possible that the extended cultivation of the Y strain in research laboratories has negatively impacted the efficiency with which it transitions to the metacyclic trypomastigote form compared to other strains.

During our longitudinal infection study, we realized that most reports using qPCR to detect *T. cruzi* in kissing bugs use total DNA extracts as templates, but a potential pitfall of this approach is the theoretical amplification of material from nonviable parasites which can inflate overall parasitemia. A recent study demonstrated that use of cDNA templates reverse transcribed from total RNA extracts is a good solution to this problem and prevents detection of nonviable parasites *in vitro* [30]. However, given additional evidence that genomic DNA from heat-killed parasites rapidly disappears in triatomines *in vivo* [29,30], we tested persistence of nonviable *T. cruzi* DNA in our *R. prolixus* culture as well. We detected short-term, strongly reduced signal in the anterior midgut that disappeared after two weeks and largely failed to detect any parasite DNA in the posterior midgut or hindgut, which we attribute to digestive enzymes (e.g., nucleases) in the posterior midgut [71]. Given that our data show the hindgut is the key tissue in which parasites stably reside, and given the greater stability of DNA during extraction compared to RNA, this data supports the continued use of qPCR using DNA templates as a suitable methodology for long-term characterization of *T. cruzi* infection kinetics in kissing bugs.

When examining infection kinetics, we were curious to know if *T. cruzi* movement through the vector intestinal tract was driven by chemotactic cues as has been observed in *T. brucei* [72] or if parasites were simply carried along with digestive contents. Using fluorescent microspheres to monitor peristalsis rate, we were surprised to find that epimastigotes, which are highly motile, appear to reach the hindgut at the same rate as the inert beads. This observation may be due to several factors: the movement of parasites might be obstructed by debris such as erythrocyte ghosts, hemozoin, and gut bacteria; parasites might only pass through gut sphincters at specific times during their dilation; or epimastigotes do not swim freely but preferentially attach to the migrating perimicrovillar membrane which moves parasites posteriorly, akin to a conveyor belt [73]. Importantly, the lack of demonstrable chemotaxis in *T. cruzi* suggests that the salivarian transmission strategy of *T. brucei* likely necessitated the development of novel mechanisms to guide movement against the natural peristaltic flow of the digestive tract and toward the insect foregut and salivary glands [74].

Overall, while our experimental infections clearly show Y strain *T. cruzi* stably colonizes our laboratory culture of *R. prolixus* under controlled conditions, an important caveat or limitation of this combination as a potential model system is that it may be a non-natural pairing [75]. As previously noted, *Rhodnius* sp. surveyed in endemic areas dominantly associate with DTU I strains of *T. cruzi* and are very seldom colonized by DTU II strains [17,20,31]. Indeed, the absence of DTU II infection in *R. prolixus* in natural settings combined with the specific trypanolytic activity of its hemolymph and saliva have led some to suggest this species may even control (restrict) DTU II geographic distribution [76]. However, a curious facet of DTU II *T. cruzi* biology is that in stark contrast with its moderate-to-high incidence in human infections (e.g., 66% in Brazil), presence of DTU II in triatomines is universally low regardless of genus or species (4%) with the exception of *Mepraia* sp. (24%) which are only found in Chile [20]. It is crucially important to study DTU II strain interactions with triatomine hosts: first, it is highly significant as a human pathogen throughout South and Central America, and second, alongside DTU I it is one of the parental lineages from which all other DTUs derive [77]. Given the infrequent detection of DTU II *T. cruzi* in insects to date, it is currently difficult to identify which triatomine genus or species likely acts as the TcII primary vector in endemic areas. We therefore propose that *R. prolixus,* as a major vector species and model organism in entomology, is at this time the ideal triatomine with which to begin DTU II-insect interaction research.

Taken together, results from our study clearly demonstrate that Y strain *T. cruzi* successfully colonizes *R. prolixus* under laboratory conditions*.* We propose that the *R. prolixus* - Y strain *T. cruzi* pairing has the potential to furnish an excellent model system with which to move forward vis-a-vis study of molecular interactions given both organisms now can be mutagenized using CRISPR/Cas9 genome editing [78]. Establishment of this system using genetically tractable model organisms opens the door to more sophisticated research directions that will allow us to untangle the functional role that both parasite and vector genes play in dictating infection and transmission outcomes of this important yet neglected disease.

## Supporting information

S1 Table Primer sequences and expected amplicon lengths for PCR to determine *T. cruzi* DTU.
Primers and PCR assay design were originally developed by Munoz-San Martin *et al.* (37).
(DOCX)

S1 Fig Confirmation of Y and Brazil strain genotypes.
PCR of genomic DNA isolated from Y and Brazil strain T. cruzi using primers specific for DTUs I-VI (37) shows unique amplification of TcII for Y strain (expected amplicon = 110 bp) and of TcI for Brazil strain (expected amplicon = 173 bp).
(TIF)

S1 File Raw data used in the study.
(XLSX)

## References

[1] Bern C, Kjos S, Yabsley MJ, Montgomery SP. Trypanosoma cruzi and Chagas’ Disease in the United States. Clin Microbiol Rev. 2011;24(4):655–81. doi: 10.1128/CMR.00005-11 21976603 PMC3194829

[2] Stanaway JD, Roth G. The burden of Chagas disease: estimates and challenges. Glob Heart. 2015;10(3):139–44. doi: 10.1016/j.gheart.2015.06.001 26407508

[3] Malik LH, Singh GD, Amsterdam EA. The Epidemiology, clinical manifestations, and management of chagas heart disease. Clin Cardiol. 2015;38(9):565–9. doi: 10.1002/clc.22421 25993972 PMC6490782

[4] Meymandi S, Hernandez S, Park S, Sanchez DR, Forsyth C. Treatment of Chagas Disease in the United States. Curr Treat Options Infect Dis. 2018;10(3):373–88. doi: 10.1007/s40506-018-0170-z 30220883 PMC6132494

[5] Bivona AE, Alberti AS, Cerny N, Trinitario SN, Malchiodi EL. Chagas disease vaccine design: the search for an efficient Trypanosoma cruzi immune-mediated control. Biochim Biophys Acta Mol Basis Dis. 2020;1866(5):165658. doi: 10.1016/j.bbadis.2019.165658 31904415

[6] Bonney KM. Chagas disease in the 21st century: a public health success or an emerging threat?. Parasite. 2014;21:11. doi: 10.1051/parasite/2014012 24626257 PMC3952655

[7] Coura JR. The main sceneries of Chagas disease transmission. The vectors, blood and oral transmissions--a comprehensive review. Mem Inst Oswaldo Cruz. 2015;110(3):277–82. doi: 10.1590/0074-0276140362 25466622 PMC4489464

[8] Nouvellet P, Dumonteil E, Gourbière S. The improbable transmission of Trypanosoma cruzi to human: the missing link in the dynamics and control of Chagas disease. PLoS Negl Trop Dis. 2013;7(11):e2505. doi: 10.1371/journal.pntd.0002505 24244766 PMC3820721

[9] Martín-Escolano J, Marín C, Rosales MJ, Tsaousis AD, Medina-Carmona E, Martín-Escolano R. An updated View of the trypanosoma cruzi life cycle: intervention points for an effective treatment. ACS Infect Dis. 2022;8(6):1107–15. doi: 10.1021/acsinfecdis.2c00123 35652513 PMC9194904

[10] Medina-Rincón GJ, Gallo-Bernal S, Jiménez PA, Cruz-Saavedra L, Ramírez JD, Rodríguez MJ, et al. Molecular and clinical aspects of chronic manifestations in Chagas disease: a state-of-the-art review. Pathogens. 2021;10(11):1493. doi: 10.3390/pathogens10111493 34832648 PMC8619182

[11] Alba Soto C, González Cappa S. Trypanosoma cruzi journey from the insect vector to the host cell. Chagas Disease: A Clinical Approach. 2019;25–59.

[12] Tarleton RL, Sun J, Zhang L, Postan M. Depletion of T-cell subpopulations results in exacerbation of myocarditis and parasitism in experimental Chagas’ disease. Infect Immun. 1994;62(5):1820–9. doi: 10.1128/iai.62.5.1820-1829.1994 8168945 PMC186416

[13] da Silva AM, Eduardo Ramirez L, Vargas M, Chapadeiro E, Brener Z. Evaluation of the rabbit as a model for Chagas disease-II. Histopathologic studies of the heart, digestive tract and skeletal muscle. Mem Inst Oswaldo Cruz. 1996;91(2):199–206. doi: 10.1590/s0074-02761996000200015 8736091

[14] Jelicks L, Tanowitz H. Advances in imaging of animal models of Chagas disease. Advances in Parasitology. 2011;75:193–208. 21820557 10.1016/B978-0-12-385863-4.00009-5 PMC3556243

[15] Mateus J, Guerrero P, Lasso P, Cuervo C, Gonzalez JM, Puerta CJ, et al. An animal model of acute and chronic Chagas disease with the reticulotropic Y Strain of Trypanosoma cruzi That depicts the multifunctionality and dysfunctionality of T cells. Front Immunol. 2019;10:918. 31105709 10.3389/fimmu.2019.00918 PMC6499084

[16] Kollien AH, Schaub GA. The development of Trypanosoma cruzi in triatominae. Parasitol Today. 2000;16(9):381–7. doi: 10.1016/s0169-4758(00)01724-5 10951597

[17] Schaub GA. An update on the knowledge of parasite–vector interactions of Chagas Disease. Research and reports in tropical medicine. 2021; 63–76. 34093053 10.2147/RRTM.S274681 PMC8169816

[18] Guarneri A, Lorenzo M. Triatominae-The biology of chagas disease vectors. Springer; 2021.

[19] de Azambuja P, Garcia E. Care and maintenance of triatomine colonies. The molecular Biology of insect disease vectors: A methods manual. Springer; 1997, p. 56–64.

[20] Brenière SF, Waleckx E, Barnabé C. Over Six Thousand trypanosoma cruzi strains classified into discrete typing units (DTUs): attempt at an inventory. PLoS Negl Trop Dis. 2016;10(8):e0004792. doi: 10.1371/journal.pntd.0004792 27571035 PMC5003387

[21] Wang W, Peng D, Baptista RP, Li Y, Kissinger JC, Tarleton RL. Strain-specific genome evolution in Trypanosoma cruzi, the agent of Chagas disease. PLoS Pathog. 2021;17(1):e1009254. 33508020 10.1371/journal.ppat.1009254 PMC7872254

[22] Peng D, Kurup SP, Yao PY, Minning TA, Tarleton RL. CRISPR-Cas9-mediated single-gene and gene family disruption in Trypanosoma cruzi. mBio. 2014;6(1):e02097-14. doi: 10.1128/mBio.02097-14 25550322 PMC4281920

[23] Soares Medeiros LC, South L, Peng D, Bustamante JM, Wang W, Bunkofske M. Rapid, selection-free, high-efficiency genome editing in protozoan parasites using CRISPR-Cas9 ribonucleoproteins. mBio. 2017;8(6):e01911-17. doi: 10.1128/mBio.01911-17 PMC5676044 29114029

[24] Lander N, Li Z-H, Niyogi S, Docampo R. CRISPR/Cas9-Induced Disruption of Paraflagellar Rod Protein 1 and 2 Genes in Trypanosoma cruzi Reveals Their Role in Flagellar Attachment. mBio. 2015;6(4):e01012. doi: 10.1128/mBio.01012-15 26199333 PMC4513075

[25] Lander N, Chiurillo M, Storey M, Vercesi A, Docampo R. CRISPR/Cas9-mediated endogenous C-terminal tagging of Trypanosoma cruzi genes reveals the acidocalcisome localization of the inositol 1,4,5-trisphosphate receptor. J Biol Chem. 2016;291(49):25505–15. 27793988 10.1074/jbc.M116.749655 PMC5207250

[26] Azambuja P, Feder D, Garcia ES. Isolation of Serratia marcescens in the midgut of Rhodnius prolixus: impact on the establishment of the parasite Trypanosoma cruzi in the vector. Exp Parasitol. 2004;107(1–2):89–96. doi: 10.1016/j.exppara.2004.04.007 15208042

[27] Castro DP, Moraes CS, Gonzalez MS, Ratcliffe NA, Azambuja P, Garcia ES. Trypanosoma cruzi immune response modulation decreases microbiota in Rhodnius prolixus gut and is crucial for parasite survival and development. PLoS One. 2012;7(5):e36591. doi: 10.1371/journal.pone.0036591 22574189 PMC3344921

[28] Vieira CS, Waniek PJ, Castro DP, Mattos DP, Moreira OC, Azambuja P. Impact of Trypanosoma cruzi on antimicrobial peptide gene expression and activity in the fat body and midgut of Rhodnius prolixus. Parasit Vectors. 2016;9:119. doi: 10.1186/s13071-016-1398-4 26931761 PMC4774030

[29] Dias F de A, Guerra B, Vieira LR, Perdomo HD, Gandara ACP, Amaral RJV do, et al. Monitoring of the parasite load in the digestive tract of rhodnius prolixus by combined qPCR analysis and imaging techniques provides new insights into the trypanosome life cycle. PLoS Negl Trop Dis. 2015;9(10):e0004186. doi: 10.1371/journal.pntd.0004186 26496442 PMC4619730

[30] Finamore-Araujo P, Silva da Fonseca GL, Vieira CS, de Castro DP, Moreira OC. RNA as a feasible marker of Trypanosoma cruzi viability during the parasite interaction with the triatomine vector Rhodnius prolixus (Hemiptera, Triatominae). PLoS Negl Trop Dis. 2022;16(7):e0010535. doi: 10.1371/journal.pntd.0010535 35797352 PMC9307183

[31] Izeta-Alberdi A, Ibarra-Cerdeña CN, Moo-Llanes DA, Ramsey JM. Geographical, landscape and host associations of Trypanosoma cruzi DTUs and lineages. Parasit Vectors. 2016;9(1):631. doi: 10.1186/s13071-016-1918-2 27923409 PMC5142175

[32] Bilheiro AB, Costa G da S, Araújo MS, Ribeiro WAR, Finamore-Araújo P, Moreira OC, et al. Detection and genotyping of Trypanosoma cruzi samples in species of genus rhodnius from different environments in the Brazilian Amazon. Vector Borne Zoonotic Dis. 2024;24(2):95–103. doi: 10.1089/vbz.2023.0082 38165392

[33] Castro DP, Seabra SH, Garcia ES, de Souza W, Azambuja P. Trypanosoma cruzi: ultrastructural studies of adhesion, lysis and biofilm formation by Serratia marcescens. Exp Parasitol. 2007;117(2):201–7. doi: 10.1016/j.exppara.2007.04.014 17570364

[34] Azambuja P, Garcia ES, Ratcliffe NA. Gut microbiota and parasite transmission by insect vectors. Trends Parasitol. 2005;21(12):568–72. doi: 10.1016/j.pt.2005.09.011 16226491

[35] Gilliland CA, Patel V, McCormick AC, Mackett BM, Vogel KJ. Using axenic and gnotobiotic insects to examine the role of different microbes on the development and reproduction of the kissing bug Rhodnius prolixus (Hemiptera: Reduviidae). Mol Ecol. 2023;32(4):920–35. doi: 10.1111/mec.16800 36464913 PMC10107482

[36] Kirchhoff LV, Hieny S, Shiver GM, Snary D, Sher A. Cryptic epitope explains the failure of a monoclonal antibody to bind to certain isolates of Trypanosoma cruzi. J Immunol. 1984;133(5):2731–5. doi: 10.4049/jimmunol.133.5.2731 6207242

[37] Muñoz-San Martín C, Apt W, Zulantay I. Real-time PCR strategy for the identification of Trypanosoma cruzi discrete typing units directly in chronically infected human blood. Infect Genet Evol. 2017;49:300–8. doi: 10.1016/j.meegid.2017.02.006 28185987

[38] Chasen NM, Coppens I, Etheridge RD. Identification and localization of the first known proteins of the Trypanosoma cruzi Cytostome Cytopharynx endocytic complex. Front Cell Infect Microbiol. 2020;9:445. doi: 10.3389/fcimb.2019.00445 32010635 PMC6978632

[39] Maddrell S. Excretion in the blood-sucking bug, Rhodnius prolixus Stål: I. The control of diuresis. Journal of Experimental Biology. 1963;40(2):247–56.

[40] Cummings KL, Tarleton RL. Rapid quantitation of Trypanosoma cruzi in host tissue by real-time PCR. Mol Biochem Parasitol. 2003;129(1):53–9. doi: 10.1016/s0166-6851(03)00093-8 12798506

[41] Moser DR, Kirchhoff LV, Donelson JE. Detection of Trypanosoma cruzi by DNA amplification using the polymerase chain reaction. J Clin Microbiol. 1989;27(7):1477–82. doi: 10.1128/jcm.27.7.1477-1482.1989 2504769 PMC267598

[42] Melo R de FP, Guarneri AA, Silber AM. The Influence of Environmental Cues on the Development of Trypanosoma cruzi in Triatominae Vector. Front Cell Infect Microbiol. 2020;10:27. doi: 10.3389/fcimb.2020.00027 32154185 PMC7046586

[43] Carmona-Peña SP, Vázquez-Chagoyán JC, Castro DP, Genta FA, Contreras-Garduño J. Benefits and costs of immune memory in Rhodnius prolixus against Trypanosoma cruzi. Microb Pathog. 2022;165:105505. doi: 10.1016/j.micpath.2022.105505 35341956

[44] Mello CB, Azambuja P, Garcia ES, Ratcliffe NA. Differential in vitro and in vivo behavior of three strains of Trypanosoma cruzi in the gut and hemolymph of Rhodnius prolixus. Exp Parasitol. 1996;82(2):112–21. doi: 10.1006/expr.1996.0015 8617337

[45] Guarneri AA, Lorenzo MG. Triatomine physiology in the context of trypanosome infection. J Insect Physiol. 2017;97:66–76. doi: 10.1016/j.jinsphys.2016.07.005 27401496

[46] Han YS, Barillas-Mury C. Implications of Time Bomb model of ookinete invasion of midgut cells. Insect Biochem Mol Biol. 2002;32(10):1311–6. doi: 10.1016/s0965-1748(02)00093-0 12225921

[47] Silva Pereira S, Trindade S, De Niz M, Figueiredo LM. Tissue tropism in parasitic diseases. Open Biol. 2019;9(5):190036. doi: 10.1098/rsob.190036 31088251 PMC6544988

[48] Van Den Abbeele J, Claes Y, van Bockstaele D, Le Ray D, Coosemans M. Trypanosoma brucei spp. development in the tsetse fly: characterization of the post-mesocyclic stages in the foregut and proboscis. Parasitology. 1999;118(Pt 5):469–78. doi: 10.1017/s0031182099004217 10363280

[49] Walsh B, Hill KL. Right place, right time: Environmental sensing and signal transduction directs cellular differentiation and motility in Trypanosoma brucei. Mol Microbiol. 2021;115(5):930–41. doi: 10.1111/mmi.14682 33434370 PMC8405151

[50] Bates PA. Transmission of Leishmania metacyclic promastigotes by phlebotomine sand flies. Int J Parasitol. 2007;37(10):1097–106. doi: 10.1016/j.ijpara.2007.04.003 17517415 PMC2675784

[51] Bargues M, Schofield C, Dujardin J-P. Classification and phylogeny of the Triatominae. American Trypanosomiasis. Elsevier; 2010. p. 117–47.

[52] Azambuja P, Garcia ES, Waniek PJ, Vieira CS, Figueiredo MB, Gonzalez MS, et al. Rhodnius prolixus: from physiology by Wigglesworth to recent studies of immune system modulation by Trypanosoma cruzi and Trypanosoma rangeli. J Insect Physiol. 2017;97:45–65. doi: 10.1016/j.jinsphys.2016.11.006 27866813

[53] Mesquita RD, Vionette-Amaral RJ, Lowenberger C, Rivera-Pomar R, Monteiro FA, Minx P, et al. Genome of Rhodnius prolixus, an insect vector of Chagas disease, reveals unique adaptations to hematophagy and parasite infection. Proceedings of the National Academy of Sciences of the United States of America. 2015;112(48):14936–41. doi: 10.1073/pnas.1511557112 26627243 PMC4672799

[54] Lander N, Chiurillo MA, Docampo R. Genome editing by CRISPR/Cas9 in Trypanosoma cruzi. T cruzi Infection: Methods and Protocols. 2019;61–76. 10.1007/978-1-4939-9148-8_5 30868519

[55] Wiedeman J, Harrison R, Etheridge RD. A limitation lifted: A conditional knockdown system reveals essential roles for Polo-like kinase and Aurora kinase 1 in Trypanosoma cruzi cell division. Proceedings of the National Academy of Sciences. 2025;122(8): e2416009122. doi: 10.1073/pnas.2416009122 PMC11874021 40106484

[56] Wang S, Dos-Santos ALA, Huang W, Liu KC, Oshaghi MA, Wei G, et al. Driving mosquito refractoriness to Plasmodium falciparum with engineered symbiotic bacteria. Science. 2017;357(6358):1399–402. doi: 10.1126/science.aan5478 28963255 PMC9793889

[57] Castro DP, Moraes CS, Garcia ES, Azambuja P. Inhibitory effects of d-mannose on trypanosomatid lysis induced by Serratia marcescens. Exp Parasitol. 2007;115(2):200–4. doi: 10.1016/j.exppara.2006.08.001 16989812

[58] Barbosa HJ, Quevedo YS, Torres AM, Veloza GAG, Carranza Martínez JC, Urrea-Montes DA, et al. Comparative proteomic analysis of the hemolymph and salivary glands of Rhodnius prolixus and R. colombiensis reveals candidates associated with differential lytic activity against Trypanosoma cruzi Dm28c and T. cruzi Y. PLoS Negl Trop Dis. 2024;18(4):e0011452. doi: 10.1371/journal.pntd.0011452 38568999 PMC10990223

[59] Ferreira RC, Kessler RL, Lorenzo MG, Paim RMM, Ferreira LDL, Probst CM, et al. Colonization of Rhodnius prolixus gut by Trypanosoma cruzi involves an extensive parasite killing. Parasitology. 2016;143(4):434–43. doi: 10.1017/S0031182015001857 26818093

[60] Suárez-Quevedo Y, Barbosa-Vinasco HJ, Gutiérrez-Garnizo SA, Olaya-Morales JL, Zabala-González D, Carranza-Martínez JC, et al. Innate trypanolytic factors in triatomine hemolymph against Trypanosoma rangeli and T. cruzi: a comparative study in eight Chagas disease vectors. Rev Acad Colomb Cienc Ex Fis Nat. 2020;44(170):88–104. doi: 10.18257/raccefyn.1097

[61] Guevara P, Dias M, Rojas A, Crisante G, Abreu-Blanco MT, Umezawa E, et al. Expression of fluorescent genes in Trypanosoma cruzi and Trypanosoma rangeli (Kinetoplastida: Trypanosomatidae): its application to parasite-vector biology. J Med Entomol. 2005;42(1):48–56. doi: 10.1093/jmedent/42.1.48 15691008

[62] Henriques C, Castro DP, Gomes LHF, Garcia ES, de Souza W. Bioluminescent imaging of Trypanosoma cruzi infection in Rhodnius prolixus. Parasit Vectors. 2012;5:214. doi: 10.1186/1756-3305-5-214 23013827 PMC3481367

[63] Pizarro J, Lucero D, Stevens L. PCR reveals significantly higher rates of Trypanosoma cruzi infection than microscopy in the Chagas vector, Triatoma infestans: high rates found in Chuquisaca, Bolivia. BMC Infectious Diseases. 2007;7(1):1–8. 10.1186/1471-2334-7-66 PMC1920523 17597541

[64] Moreira OC, Verly T, Finamore-Araujo P, Gomes SAO, Lopes CM, de Sousa DM, et al. Development of conventional and real-time multiplex PCR-based assays for estimation of natural infection rates and Trypanosoma cruzi load in triatomine vectors. Parasit Vectors. 2017;10(1):404. doi: 10.1186/s13071-017-2343-x 28851417 PMC5576278

[65] Schijman AG. Molecular diagnosis of Trypanosoma cruzi. Acta Trop. 2018;184:59–66. doi: 10.1016/j.actatropica.2018.02.019 29476727

[66] Alves CR, Albuquerque-Cunha JM, Mello CB, Garcia ES, Nogueira NF, Bourguingnon SC, et al. Trypanosoma cruzi: attachment to perimicrovillar membrane glycoproteins of Rhodnius prolixus. Exp Parasitol. 2007;116(1):44–52. doi: 10.1016/j.exppara.2006.11.012 17250827

[67] Lemos JRD, Rodrigues WF, Miguel CB, Parreira RC, Miguel RB, de Paula Rogerio A, et al. Influence of parasite load on renal function in mice acutely infected with Trypanosoma cruzi. PLoS One. 2013;8(8):e71772. doi: 10.1371/journal.pone.0071772 23951243 PMC3741127

[68] Asin S, Catalá S. Development of Trypanosoma cruzi in Triatoma infestans: influence of temperature and blood consumption. J Parasitol. 1995;81(1):1–7. 7876960

[69] Schaub GA. Chapter 4 Interactions of Trypanosomatids and Triatomines. Advances in Insect Physiology. 2009;177–242. doi: 10.1016/s0065-2806(09)37004-6

[70] Ramos LG, de Souza KR, Júnior PAS, Câmara CC, Castelo-Branco FS, Boechat N, et al. Tackling the challenges of human Chagas disease: A comprehensive review of treatment strategies in the chronic phase and emerging therapeutic approaches. Acta Trop. 2024;256:107264. doi: 10.1016/j.actatropica.2024.107264 38806090

[71] Oliveira PL, Genta FA. Blood digestion in triatomine insects. Triatominae-The Biology of Chagas Disease Vectors. Springer; 2021. p. 265–84.

[72] DeMarco S, Saada E, Lopez M, Hill K. Identification of positive chemotaxis in the protozoan pathogen Trypanosoma brucei. mSphere. 2020;5(4). 10.1128/mSphere.00685-20 PMC7426175 32817459

[73] Bittencourt-Cunha PR, Silva-Cardoso L, Oliveira GA de, Silva JR da, Silveira AB da, Kluck GEG, et al. Perimicrovillar membrane assembly: the fate of phospholipids synthesised by the midgut of Rhodnius prolixus. Mem Inst Oswaldo Cruz. 2013;108(4):494–500. doi: 10.1590/S0074-0276108042013016 23827998 PMC3970613

[74] Bachmaier S, Giacomelli G, Calvo-Alvarez E, Vieira LR, Van Den Abbeele J, Aristodemou A, et al. A multi-adenylate cyclase regulator at the flagellar tip controls African trypanosome transmission. Nat Commun. 2022;13(1):5445. doi: 10.1038/s41467-022-33108-z 36114198 PMC9481589

[75] Tripet F. Ecological immunology of mosquito-malaria interactions: Of non-natural versus natural model systems and their inferences. Parasitology. 2009;136(14):1935–42. doi: 10.1017/S0031182009006234 19490728

[76] Vallejo G, Barbosa H, Suarez Y, Meneses A, Carranza J, Urrea D. La respuesta inmune de algunas especies del género Rhodnius modifica la circulación de genotipos de Trypanosoma cruzi y T. rangeli en varias regiones de América Latina. Revista de la Academia Colombiana de Ciencias Exactas, Físicas y Naturales. 2023;47(185):765–84.

[77] Zingales B, Macedo AM. Fifteen Years after the Definition of Trypanosoma cruzi DTUs: What Have We Learned?. Life (Basel). 2023;13(12):2339. doi: 10.3390/life13122339 38137940 PMC10744745

[78] Lima L, Berni M, Mota J, Bressan D, Julio A, Cavalcante R, et al. Gene Editing in the Chagas Disease Vector Rhodnius prolixus by Cas9-Mediated ReMOT Control. CRISPR J. 2024;7(2):88–99. doi: 10.1089/crispr.2023.0076 38564197

