# Supplementary material for: Stable colonization of the kissing bug Rhodnius prolixus by Trypanosoma cruzi Y strain
Source: PLoS Negl Trop Dis. 2025 Mar 12;19(3):e0012906. doi: 10.1371/journal.pntd.0012906 (PMC11928063; doi:10.1371/journal.pntd.0012906)
Supplement: S1 Table — Primers and PCR assay design were originally developed by Munoz-San Martin et al. (37). (DOCX) [file pntd.0012906.s001.docx]

| **DTU** | **Target Gene** | **Primer Sequence (5’ ̵ 3’)** | **Amplicon (bp)** |
| --- | --- | --- | --- |
| **TcI** | *SL-IR* | Fd: GCTTTGTGTTCTCGCACTCCA  Rv: CGATCAGCGCCACAGAAAGT | 173 |
| **TcII** | *COII* | Fd: GGATTACATCTACGGCTGACACC  Rv: CGAGAGTGATTATTTGGTGGGAGATA | 110 |
| **TcIII** | *SL-IR* | Fd: TGCGCCCGTGTTGTTGTTC  Rv: CGGTGCATACACGCGATTCC | 161 |
| **TcIV** | *COII* | Fd: TGAATGAATGACATCTACAGCG  Rv: TATTTGGAGAGACTACAATATTTAGT | 152 |
| **TcV** | *ND1* | Fd: AGTTTTATTAATCTTATCAGGATTTGGTG  Rv: CCATCTGTGATAGGTGTTAATATTCC | 120 |
| **TcVI** | *18S rDNA* | Fd: CGTAGGCGTGGTCGGGT  Rv: TATTCCCGTTAAAGGCCCTTGT | 132 |

**S1 Table. Primer sequences and expected amplicon lengths for PCR to determine *T. cruzi* DTU.**
