# Supplementary figures and images for: Stable colonization of the kissing bug Rhodnius prolixus by Trypanosoma cruzi Y strain
Source: PLoS Negl Trop Dis. 2025 Mar 12;19(3):e0012906. doi: 10.1371/journal.pntd.0012906 (PMC11928063; doi:10.1371/journal.pntd.0012906)

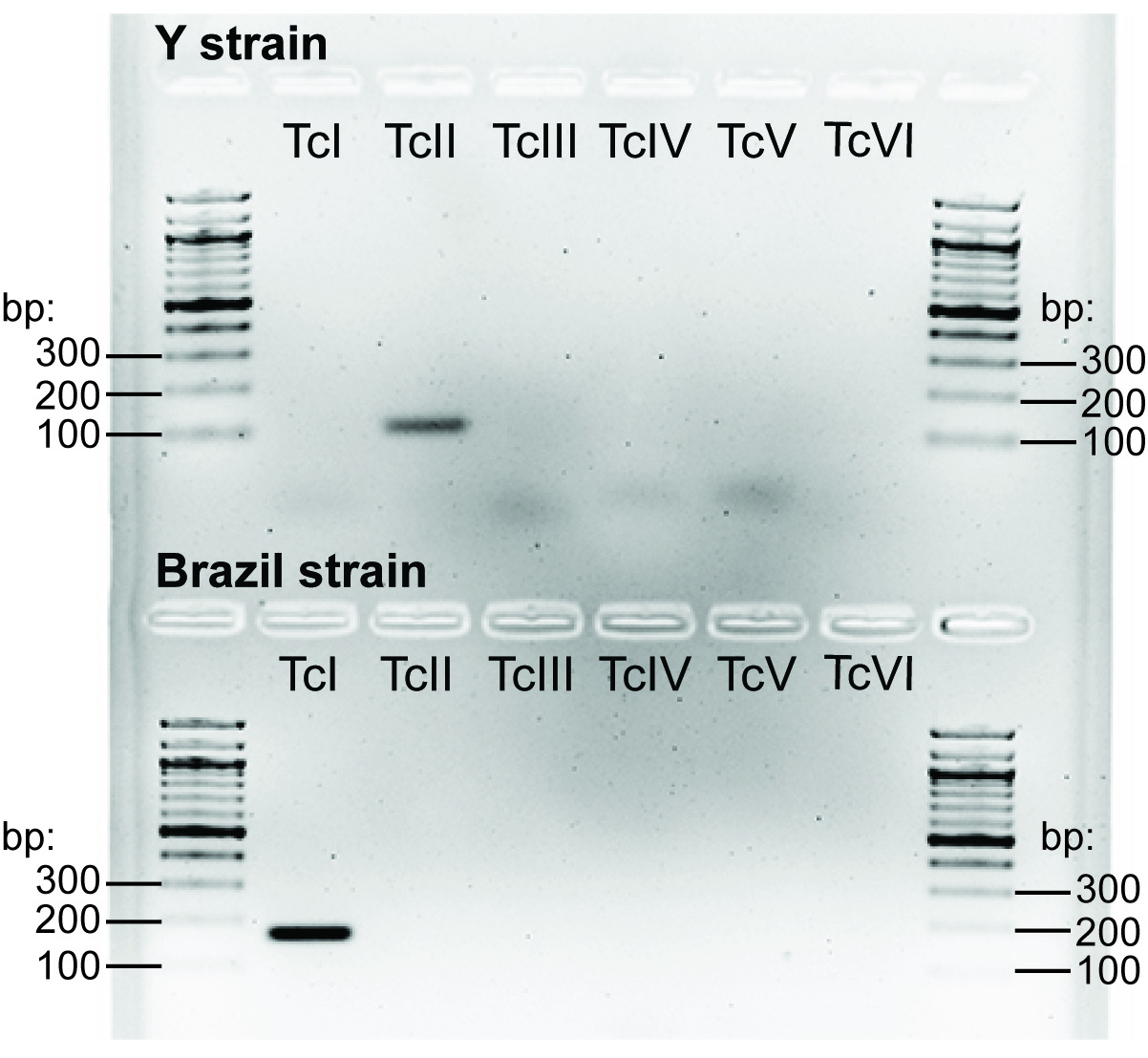

Supplement: S1 Fig — PCR of genomic DNA isolated from Y and Brazil strain T. cruzi using primers specific for DTUs I-VI (37) shows unique amplification of TcII for Y strain (expected amplicon = 110 bp) and of TcI for Brazil strain (expected amplicon = 173 bp). (TIF) [file pntd.0012906.s002.tif]
